# Is motivation enough? Responsiveness, patient-centredness, medicalization and cost in family practice and conventional care settings in Thailand

**DOI:** 10.1186/1478-4491-4-19

**Published:** 2006-07-27

**Authors:** Yongyuth Pongsupap, Wim Van Lerberghe

**Affiliations:** 1 National Health Security Office, Nonthaburi, Thailand; 2 Department of Health Policy, Development and Services; World Health Organization, Geneva, Switzerland

## Abstract

**Background:**

In Thailand, family practice was developed primarily through a small number of self-styled family practitioners, who were dedicated to this professional field without having benefited from formal training in the specific techniques of family practice. In the context of a predominantly hospital-based health care system, much depends on their personal motivation and commitment to this area of medicine. The purpose of this paper is to compare the responsiveness, degree of patient-centredness, adequacy of therapeutic decisions and the cost of care in 37 such self-styled family practices, i.e. practices run by doctors who call themselves family practitioners, but have not been formally trained, and in 37 conventional public hospital outpatient departments (OPDs), 37 private clinics and 37 private hospital OPDs.

**Method:**

Analysis of the characteristics of 148 taped consultations with simulated patients.

**Results:**

The family practices performed better than public hospital OPDs with regard to responsiveness, patient-centredness and cost of technical investigations (M-W U: p < 0.001). Prescribing patterns were similar, but family practices prescribed fewer drugs and were less costly than private clinics and hospitals (M-W U: p < 0.001). The degree of patient-centredness was not significantly different. Private clinics and private hospitals scored better for responsiveness.

**Conclusion:**

In Thailand self-styled family practices, even without specific training, provide a service that is more responsive and patient-centred than conventional care, with less overmedicalization and at a lower cost. Changes in prescription practices may require deeper changes in the medical culture.

## Introduction

Family practice is a new concept in Thailand. The (modern) Thai health care system is essentially based upon biomedical and hospital-centred care and has been so since its introduction, at the end of the nineteenth century. Both supply and demand are dominated by a reliance on technology and specialization: health care is essentially a commodity [1,2].

Over the last 10 years there have been attempts to develop family practice in Thailand. This was largely a reaction against the lack of emphasis on the human dimension of health care. However, family practice is also supposed to address a number of other issues: to improve responsiveness, encourage patient-centred consultation, decrease unnecessary prescriptions, control costs and improve patient satisfaction. Attempts to develop the discipline of family practice in Thailand have largely been an initiative of public sector doctors, also because these saw it as a way to attract patients, who tend to prefer going to specialists in the private sector.

One cannot yet speak of a fully developed professional identity for family practitioners. The College of Family Practitioners was created only in 1999, and formal training is just beginning. Nevertheless, a number of self-styled family practitioners started operating during the late 1990s, within a loose network of doctors who had attended a variety of training courses and internships. The prime movers of this initiative obtained official recognition of their family practice by the Ministry of Public Health.

This paper examines whether the self-styled family practices launched in the 1990s fulfil the expectations of family practice by looking at responsiveness, degree of patient-centredness, prescription habits and costs, through a simulated (or "dummy") patient survey. This makes it possible to compare the performance of 37 of the highly motivated, original self-styled family practices with providers of conventional (public and private) outpatient care.

This is timely, as family practice has been assigned a pivotal role in financing and organization within the newly reformed Thai health care system [3]. Since then, the number of family practices has increased exponentially. This was not done, however, on the basis of a corpus of skills, competences and professional identity, but as a way to conform to administrative requirements.

## Background and methods

After appropriate training, six simulated patients (three females and three males, averaging 25 years of age) were asked to attend consultations with standardized complaints of anxiety, presenting as recurring stomach-ache that responded well to self-administered anti-acids. The current episode was said to have started four days previously. The "patients" were instructed to indicate that the problem had started four months previously, when the patient's mother had suffered a stroke. They were instructed to appear anxious, to express a fear of cancer and to request information and explanation via agreed-upon cue questions and statements. At the end of the consultation patients were instructed to ask for a prescription of sleeping pills on behalf of their (absent) mother.

The six simulated patients consulted randomly chosen doctors in each of the 37 family practices in 16 provinces, all of which were officially endorsed by the Ministry of Public Health in March 2001. These were all public facilities: 31 health centres and six family practices located within hospital OPDs. These 37 practices were compared with outpatient consultations in 37 public hospitals, 37 private clinics and 37 private hospitals in the same provinces (simple random choice). The simulated patients discreetly taped the consultation, and took structured notes afterward. The material was transcribed and handed over to the investigators after eliminating all references that would have allowed identification of the doctors concerned, for reasons of confidentiality. Approval of the research protocol and of the confidentiality procedure was earlier obtained from the relevant authorities. The different settings were compared with regard to the following dimensions: responsiveness, degree of patient-centredness, medicalization and cost.

First, the degree of responsiveness was analysed by looking at opening hours, waiting time, consultation time (component parts of which were: physical examination, dialogue with the patient and time allowed for the patient to express their concerns), request by practitioner for a review consultation and the use of the "politeness particles" "khrap" and "kaa" by the doctor. A specificity of Thai language is the use of these particles, the frequency of which, per unit of time of speech, gives a good indication of the degree of courtesy and respect shown.

Second, the degree of patient-centredness was measured by scoring responses to requests for information, empathy and anxiety relief [4]. Responses to requests for information were assessed by scoring the answers to "What is this illness?" Responses to requests for empathy with the patient's predicament were assessed by scoring the answers to: "I am under a lot of stress, I have to care for my mother who had a stroke, how can I handle all this?" Responses to requests for anxiety relief were assessed by scoring the doctors' reactions to the questions: "Why does this happen to me? Is this a cancer like my uncle had four years ago?" and "Will I die?"

The cue questions were short, in order to increase the probability of patients' being able to express these concerns during the consultations. Scoring of the transcribed tape-recordings used the following scale:

0: there was no opportunity to express the cue question or hint;

1: the doctor ignored or cut the question short,

2: the doctor responded in a closed fashion, e.g. Q: "Why does this happen to me?" A: "This can happen to anybody [followed by change of subject]";

3: the patient was allowed to elaborate;

4: the patient was encouraged to elaborate and express expectations or feelings.

This gives a possible range of 0 to 16: 0–4 for information-giving, 0–4 for empathy and 0–8 for anxiety relief.

Scoring of the tapes was performed blindly, i.e. without information on where the consultation took place (family practice, clinic, hospital and public or private). The reproducibility of the scoring method was assessed by comparing the assigned scores with those made by an independent scorer, not involved in the research, on 116 questions in 29 out of 148 consultations (systematic 1/5 sample). There was disagreement on the score in 3 out of 116 items.

The third dimension for comparison was the degree of medicalization. This was assessed by looking at: the number of drugs prescribed that were not specifically indicated by the symptoms; the number of recommended investigations; the reaction to requests for sleeping pills; and the content of the information provided to the patient.

Finally, costs were compared. The direct costs to the patient were calculated from the consultation fees plus the costs of the drugs prescribed (in Thailand these are often combined into a single fee). The simulated patients obviously did not undergo the examinations recommended by the doctors, but it was easy to cost these at the current market rates. Suggested follow-up consultations were not included. The cost to the State was extrapolated from existing costing studies.

## Results

### Responsiveness

Only 22% of the self-styled family practitioners conducted consultations outside "normal" working hours, as compared to 54% of practitioners in public facilities (all private practitioners, whether in clinics or hospitals, conducted consultation in the early morning or in the evening).

**Figure 1:**
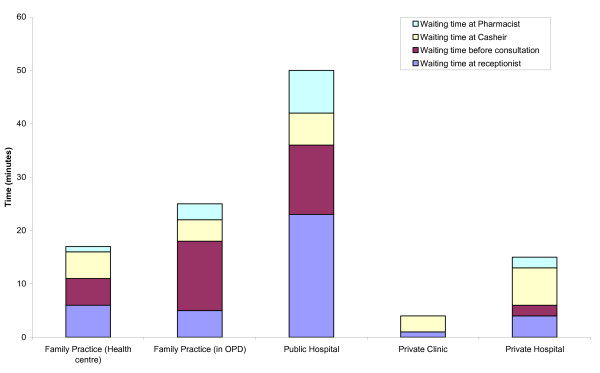
Median waiting times at the reception, before consultation, at the cashier and medication collection, for family practices and non-family practices.

Waiting times at all stages (including at reception, between reception and consultation, at the cashier following consultation and to obtain medication) were much longer in public hospitals than in family practices: an average of 71 minutes (median 83) against 36 (median 28, M-W U: p < 0.001). However, private hospitals and clinics had even shorter waiting times, averaging 23 minutes (median 18, M-W U: p = 0.30) and 18 minutes (median 11, M-W U: p < 0.001) respectively (Figure 1).

**Figure 2:**
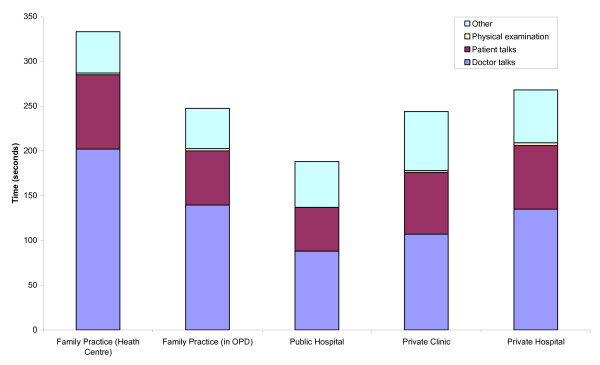
Median consultation time in family practice and non-family practice settings, disaggregated for the time patients are allowed to express their problem, the time allocated to physical examination, the time the doctor is talking to the patient, and the time the doctor spends writing or dealing with the nursing and administrative staff.

Consultation time was longer in family practices (average 6.2 minutes) when compared to private clinics (5.9 minutes), private hospitals (5.7 minutes), or public hospitals (3.8 minutes; M-W U: p < 0.001). Figure 2 shows that in all settings physical examination was perfunctory at best. Most of the difference seen in duration of the consultation was accounted for by time allowed for conversation. The median time that doctors in family practice talked to their patients was 1 minute 20 seconds, as opposed to 49 seconds for conventional doctors in public hospitals (M-W U: p < 0.001). Patients were also found to have more time to express themselves in family practice than in public hospitals, private clinics and private hospitals: average of 86 seconds against 55 seconds, 76 seconds and 80 seconds, respectively.

Patients were asked to return for a follow-up consultation in 89% of private hospital consultations, by 54% of public hospital doctors and family practice practitioners, and by 38% of private clinic doctors. Requests for follow-up visits were often related to recommendations for further technical investigations.

The use of the particles "khrap" and "kaa" (whose frequency in Thai conversation expresses courtesy and respect), shows private doctors to be the most polite (2.47 particles per minute both in private clinics and hospitals), followed by the family practitioners (2 particles per minute). Conventional public hospital doctors were found to be less polite (1.3 particles per minute).

### Patient-centredness

**Figure 3:**
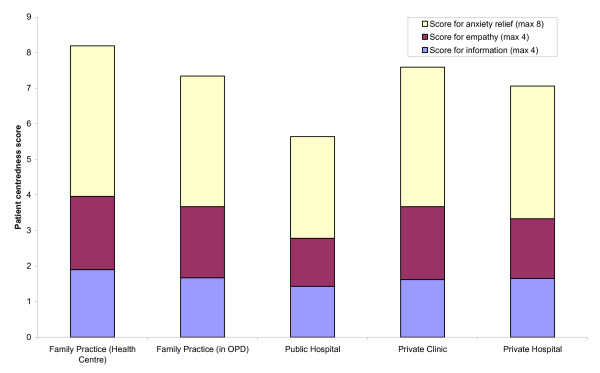
Average of patient-centredness scores in different public and private settings. The total patient-centredness score (out of a maximum of 16) is the sum of the scores for response to requests for information, for empathy and for anxiety relief

Figure 3 shows the average scores, in the various settings, of the responses to requests for information, empathy and degree of reassurance offered to ease anxiety. The "patients" tried hard to use all four sets of cue questions or statements. They succeeded in doing so in 78% of family practices, 70% of OPDs in public hospitals, 75% of private clinics and 81% in OPDs of private hospitals.

When patients were able to express the cue question or request, the most common reactions observed were either no reaction at all (score 1) or an answer that excluded further elaboration (score 2). For example, an answer to "Why is this happening to me?" may be "It's not only you, anyone can suffer from the same disease [change of subject]", or when asking "Will I die?" one reply was "Everyone dies, nobody knows when [change of subject]."

Open answers (scored 3) or encouragement of the patient to express expectations or feelings (score 4) were rare. Only 8.9% of family practices, 2.2% of public hospitals, 7.0% of private clinics and 5.1% of private hospitals reached a patient-centred approach score of 10 or more out of a maximum of 16. The median of total score for all three dimensions of patient-centredness (information-giving, empathy and anxiety relief) in family practices was 8.05/16; in public hospitals was 5.65/16 (M-W U: p < 0.001), in private clinics was 7.59/16, and 7.05/16 in private hospitals.

The opportunity allowed to "patients" to express themselves explains 36.3% of the variation in patient-centred approach scores in a regression analysis; combined with the 47% in total consultation time variation previously explained.

### Medicalization

With the type of complaint presented by the simulated patients, anxiety relief through counselling or treatment with anti-acids would be the treatments of choice. Other medications can therefore be considered as medicalization, and all of the 148 doctors overprescribed on this basis. They prescribed an average of 2.73 different drugs per patient seen in family practice, 2.68 in public hospitals, 3.41 in private clinics and 3.41 in private hospitals. Per patient, along with an average of l.4 anti-acids per patient, doctors prescribed an average of 0.6 GI regulators and 0.6 antispasmodics. Of the total, 19% of public and 27% of private doctors prescribed tranquillizers. Some private doctors also prescribed antibiotics or antidepressants; one prescribed medication to be administered by injection.

**Figure 4:**
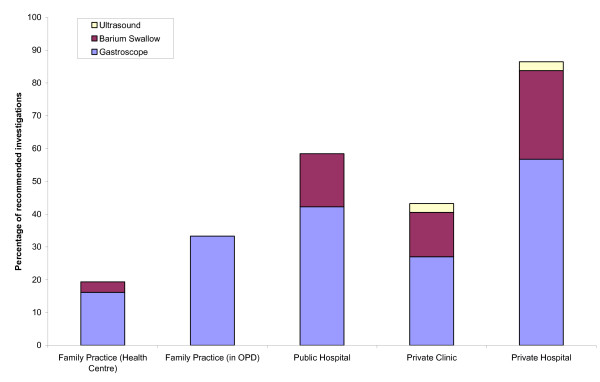
Proportion of patients to whom the doctor recommended technical investigations in different settings.

**Figure 5:**
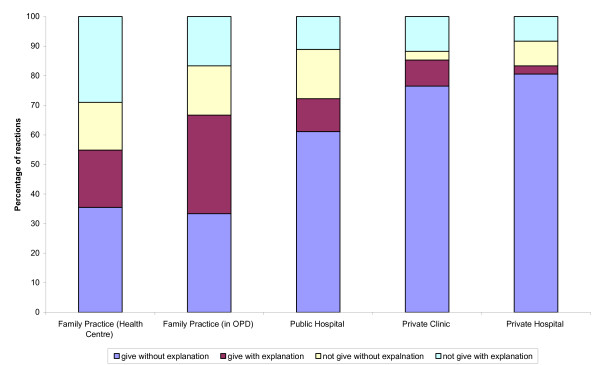
Percentage of doctors demonstrating the different reactions to a request for sleeping pills for patient's mother.

76% of the doctors in private hospitals and 41% of those in conventional public hospital OPDs recommended a endoscopy and/or barium meal investigation, compared to 32% in private clinics and 19% in family practices (Figure 4). Family practices were also the least likely to agree to requests for sleeping pills for the (absent) mother of the "patient": in two thirds of cases they would refuse or insist on seeing the person for whom the prescription was intended (Figure 5). In contrast, 76% of private clinic and 81% of private hospital doctors prescribed the drug without even asking to see the "patient's" mother.

### Cost

On the average, private hospitals charged a consultation fee of USD 1.31. Drug charges were highest in the private hospitals (USD 7.40) and lowest in the family practices (USD 1.90). As is customary in Thailand, private clinics included the consultation fee in the fee paid for the drugs (USD 5.50). Family practitioners and conventional doctors in public hospital OPDs only exceptionally charged consultation fees.

A significant part of the total cost to the patient resulted from the recommended additional technical investigations. The cost of the suggested investigations was highest in private hospitals (average USD 37.70), and lowest in family practices in health centres (average USD 3.20).

The total cost to the patient, i.e. the sum of consultation fee, drug cost and cost of recommended investigations, was highest in private hospitals (average USD 46.40), and lowest for consultations with family practitioners operating within health centres (average USD 5.20) or public hospital OPDs (average USD 6.60). This corresponds to between 1.4 and 12.5 times Thailand's minimum daily wage of USD 3.70.

The estimated unit cost to the State of a consultation in an OPD at a public hospital is within the range of USD 5.30 to USD 6.60 [5] and that of a consultation with a family practitioner at USD 1.40, whether in a health centre or in a hospital OPD [6]. This cost is not carried over to the patient.

**Figure 6:**
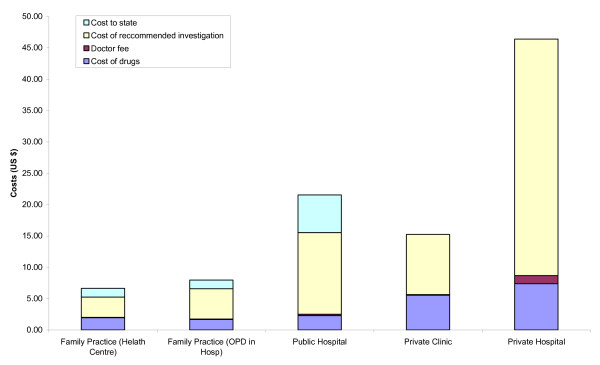
Average direct costs to the patient (consultation fees, drugs and recommended investigations) and estimated costs to the State of a medical outpatient consultation in the different settings. Note that in private clinics consultation fees are included in the drug charges.

Adding the cost to the State to the cost borne by the patient puts the total average cost of a consultation with family practitioners at about half of that of a private clinic and one third of a consultation with a conventional doctor in a public hospital OPD, and less than one sixth of a consultation in a private hospital (Figure 6).

## Discussion and conclusion

To our knowledge, this is the first comparison of family practice and non-family practice outpatient care based on the observation of a range of parameters covering responsiveness, degree of patient-centred approach, medicalization and cost. Although these various dimensions have been operationalized within the specific context of medical consultations in Thailand, this operationalization possesses face validity.

The use of simulated patients made it possible to provide first-hand, direct information on various aspects of patient care with minimal observation bias (a direct development from our earlier work on public private differences [7]). Standardized "patient" histories and cue questions, in combination with blinded analysis of the consultation tapes and transcripts, allowed for a reproducible and objective comparison of the patient-doctor interaction in the various clinical settings – with the limitations due to the choice of one single complaint presented by a first-time patient.

There is a worrisome discrepancy between the label of "family practice" as it is understood internationally and the reality of day-to-day work of these self-styled "family practitioners" in Thailand, as evidenced by the prevalence of overmedicalization and the disappointingly low degree of patient-centredness. Still, they seem to outperform conventional practice in the OPDs of public hospitals across the board; they also seem to outperform private clinics and hospitals in terms of patient-centredness, with less medicalization and at a lower cost, but not with better scores in terms of responsiveness.

Responsiveness is indeed a problem within the Thai health service, and particularly in public facilities. Family practices perform somewhat better than conventional public facilities, but there still remains room for improvement in terms of opening hours, waiting times and courtesy.

Consultations by self-styled family practitioners are longer and more patient-centred than those in other facilities. This is particularly the case for family practices located in health centres. Their score for patient-centredness was almost twice that seen in the public hospital OPDs (the conventional way of providing medical outpatient care in public facilities in Thailand). This is most likely the result of the motivation of the self-styled family doctors themselves and the user-friendly context of the health centre's environment. It has not been possible to determine to what extent the various elements of training and self-study have contributed, and to what extent self-selection and motivation explain the relatively better performance.

Nevertheless, motivation and a new working environment alone were clearly not enough to encourage doctors to go beyond a nosological categorization of the simulated patients in terms of "gastritis stress-eating behaviour". In this aspect these self-styled family practitioners behaved like most other Thai doctors. They categorized patients at a very early stage of the consultation, at the expense of listening. In Thai culture, patients are expected to pay respectful attention to what the doctor says; often cue questions could be asked only after this categorization process had already occurred.

Many doctors, in all the studied groups, seem to have recognized anxiety as a key feature of their patient's history. They did not, however, respond with information, reassurance or counselling. Rather, private practitioners, within clinics and hospitals, relied mainly on tranquilizers, but were by no means the exception, as this behaviour was also seen among family doctors. Doctors in private hospitals were not only the ones to prescribe most drugs to relieve anxiety and fear, but also more commonly recommended further investigations to be carried out. Moreover, when a patient asked for sleeping pills on behalf of the mother, the reaction of many private doctors was to provide them immediately without any further exploration.

Whether formal family practice training would be sufficient to overcome the biomedical bias prevalent among Thai doctors is a matter of speculation. Thai doctors, including those engaged in family practice, tend to over-rely on a biomedical interpretation of their patient's problems. Also, all the doctors in the study made potentially iatrogenic prescriptions of a multitude of inappropriate drugs.

This overuse of drugs and investigations does not seem to be a question of financial incentives alone. In public hospitals, doctors recommended expensive investigations in almost half of the consultations, without any personal financial reason to do so. They did so more frequently than the family practices in the health centres, and surprisingly, more than in the private clinics. It is likely that the biotechnological bias of the professional environment is an important factor for overuse and reliance upon these investigations. In health centre family practices and in clinics, where the technology was not directly available, and as there was little to be gained from recommending endoscopy and barium meal investigation, patients were less likely to be advised to undergo these complementary investigations.

The dominance of biotechnology, which is only partly compensated for by present attempts at reorienting health care delivery towards family medicine, results in inefficiency, a reduction in the degree of patient-centred approach and potentially harmful prescribing. This also has major consequences for the cost of health care for patients. If one can extrapolate the way of operating of the prime movers who are trying to introduce family practice in Thailand, it is possible to improve responsiveness and patient-centredness.

The fact is that for the same cost – both for the patient and in reduced total costs for the State – curative consultations are possible, and this should certainly be an incentive for policy-makers to support a more prominent role for family practice within the Thai health care system. It remains to be seen whether further formalization of training and accreditation of family practice will allow the average Thai doctor to do as well as or better than the prime movers of the 1990s.

## Competing interests

The author(s) declare that they have no competing interests.

## Authors' contributions

Both authors jointly carried out design, organization of the survey, data analysis and write-up of the paper. Dr Yongyuth Pongsupap analysed and scored the tape recordings.

The named authors alone are responsible for the views expressed in this article, which do not necessary reflect the position of the organizations they work for.
